# A Comparison Study on Polysaccharides Extracted from *Citrus reticulata* Blanco cv. Tankan Peel Using Five Different Methods: Structural Characterization and Immunological Competence

**DOI:** 10.3390/polym17182554

**Published:** 2025-09-22

**Authors:** Jinming Peng, Guangwei Chen, Ziyuan Lin, Shaoxin Guo, Yue Zeng, Qin Wang, Wenhua Yang, Jun Li

**Affiliations:** 1 Guangdong Key Laboratory of Science and Technology of Lingnan Specialty Food, Zhongkai University of Agriculture and Engineering, Guangzhou 510225, China; pengjmiyz@163.com (J.P.); chengw196811@163.com (G.C.); linzy20010225@163.com (Z.L.); shaoxin202407@163.com (S.G.); zy2690372544@163.com (Y.Z.); qwang1230@126.com (Q.W.); 2 Key Laboratory of Green Processing and Intelligent Manufacturing of Lingnan Specialty Food, Ministry of Agriculture, Zhongkai University of Agriculture and Engineering, Guangzhou 510225, China; 3 Key Laboratory of Processing and Nutritional Health of Tropical and Subtropical Aquatic Specialty Foods, Zhongkai University of Agriculture and Engineering, Guangzhou 510225, China

**Keywords:** *Citrus reticulata* Blanco cv. Tankan peel polysaccharides, extraction methods, structural characteristic, immunological activity

## Abstract

This is the first work to screen an optimal extraction method for *Citrus reticulata* Blanco cv. Tankan peel polysaccharides (CPP). The CPP was extracted using hot water extraction (HWE), acid extraction (AAE), enzyme extraction (EAE), high-pressure extraction (HPE), and ultrasound extraction (UAE), named CPP-W, CPP-A, CPP-E, CPP-P, and CPP-U, respectively. Results showed that CPP-A and CPP-P had higher extraction yields than other CPPs. The five CPPs varied chemically in molecular weight, monosaccharide composition, and microstructure, but shared similar IR spectra and core glycosidic linkages, indicating differential degradation while preserving core structures during extraction. Among these CPPs, CPP-A, CPP-E, and CPP-U exhibited stronger immunological activities, attributed to high galacturonic acid and low molecular weight. Moreover, CPPs significantly promoted secretion of cytokines (nitric oxide, NO; prostaglandin E_2_, PGE_2_; interleukin-6, IL-6; tumor necrosis factor-α, TNF-α) by activating downstream inducible nitric oxide synthase (iNOS) and cyclooxygenase-2 (COX-2)-related mitogen-activated protein kinases (MAPK) pathways. Overall, CPP-E possessed high extraction yield, low molecular weight, and strong immuno-stimulatory activity, suggesting that enzyme-assisted extraction was the optimal approach for extracting CPP.

## 1. Introduction

Polysaccharides, as a class of bioactive macromolecules widely distributed in plants, fungi, and animals, have emerged as a research focus owing to their diverse biological activities and few side effects [1]. Among natural sources, citrus peel stands out as a particularly promising reservoir of functional polysaccharides, owing to its abundance, low cost, and high polysaccharide content (comprising up to 80% of its total soluble components) [2,3]. *Citrus reticulata* Blanco cv. Tankan, a premium citrus cultivar, has been grown in China for thirteen centuries [4]. The peel of *Citrus reticulata* Blanco cv. Tankan, a dominant processing byproduct, has traditionally been discarded as waste or utilized as feed, manure, and biofuel [5]. Therefore, utilization of *Citrus reticulata* Blanco cv. Tankan fruit peel to extract polysaccharides represents a significant strategy toward agricultural sustainability.

The selection of an appropriate extraction method is pivotal in determining both the yield and biological activity of polysaccharides [6]. The conventional hot water extraction (HWE) has long been employed to isolate polysaccharides, but emerging techniques, including acid-assisted extraction (AAE), enzyme-assisted extraction (EAE), high-pressure extraction (HPE), and ultrasound-assisted extraction (UAE), are increasingly being adopted for their efficiency and ability to preserve structural integrity [7]. These techniques differ in their mechanisms of disrupting cell walls and solubilizing polysaccharides, potentially leading to variations in glycosidic linkage pattern, chain conformation, and functional group distribution [8]. Polysaccharides from yam peel [9] and potato peel [10] using different extracting methods presented diverse molecular weight, monosaccharide composition, glycosidic bond, and morphology, therefore contributing to their hypoglycemic and antioxidative capacities. However, to date, no information is available on the association with the *Citrus reticulata* Blanco cv. Tankan peel polysaccharide (CPP) extraction methods, characterizations, and immunological activities.

Therefore, this study aimed to extract CPP by using different extraction methods, including HWE, AAE, EAE, HPE, and UAE, and to investigate the influences of extracting strategies on yield, chemical component, molecular weight, monosaccharide composition, morphology, and structural characteristics of CPP. Furthermore, this study evaluated the in vitro immunological activities of the CPP. The obtained results will lay a scientific basis for utilizing CPP in functional foods.

## 2. Materials and Methods

### 2.1. Materials

*Citrus reticulata* Blanco cv. Tankan peel was purchased from Raoping County Huizhong Fruit and Vegetable Co., Ltd. (Chaozhou, China) in January 2023. Galacturonic acid, dextran standards, and monosaccharide standards were purchased from Merck KGaA (Darmstadt, Germany). Fetal bovine serum (FBS), penicillin-streptomycin, Dulbecco’s Modified Eagle Medium (DMEM) were acquired from Gibco (Waltham, MA, USA). 3-(4,5-Dimethyl-2-Thiazolyl)-2,5-Diphenyl Tetrazolium (MTT) and lipopolysaccharide (LPS) were sourced from Yuanye Bio-Technology (Shanghai, China). All analytical-grade chemicals were sourced from Sinopharm Chemical Reagent Co., Ltd. (Shanghai, China).

### 2.2. Preparation of Five Different CPPs

**Figure 1:**
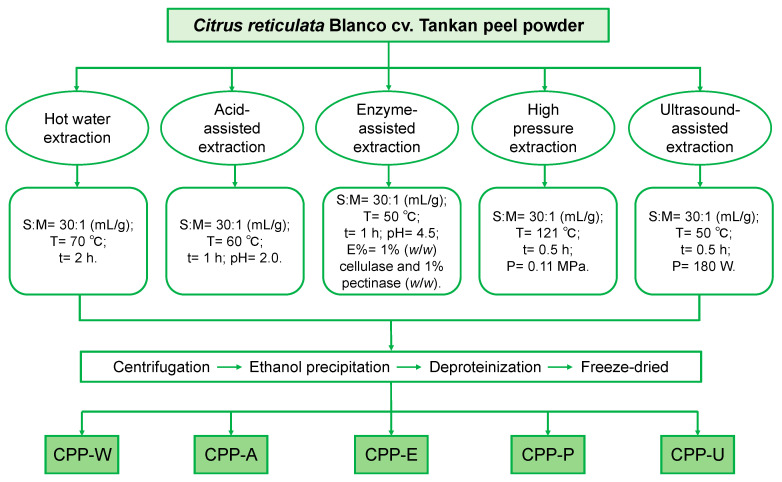
Processing of extraction of Citrus reticulata Blanco cv. Tankan polysaccharides (CPPs) by five different methods.

Fresh *Citrus reticulata* Blanco cv. Tankan peel was freeze-dried, ground into powder, then sieved through 80-mesh screens. The peel powder underwent reflux extraction using 95% aqueous ethanol (*v*/*v*) for 3 h to eliminate some pigmented components and small-molecular-weight substances, and then refluxed with petroleum ether for 2 h to remove grease. Figure 1 details the operational workflows for five extraction methods. Briefly, the hot water extraction (HWE) was performed with a solvent–material ratio of 30:1 (mL/g) at 70 °C for 2 h. The acid-assisted extraction (AAE) protocol for CPPs included solvent (0.1 M HCl aqueous solution)–material ratio 30:1 (mL/g), extracting temperature 60 °C, extracting time 1 h, and pH 2.0. The enzyme-assisted extraction (EAE) conditions included solvent–material ratio, 30:1 (mL/g); extracting temperature, 50 °C, extracting time, 1 h; solvent, 1% (*w*/*w*) cellulase and 1% (*w*/*w*) pectinase in acetate buffer (pH 4.5). Subsequently, the extract was heated at 95 °C for 10 min to inactivate the enzymes. For high-pressure extraction (HPE), the sample solution was placed in a high-pressure (0.11 MPa) steam sterilization pot (Qingdao Jingcheng Medical Instrument Factory, Qingdao, China) with a solvent (distilled water)–material ratio of 30:1 (mL/g) at 121 °C for 0.5 h. The ultrasound-assisted extraction (UAE) was conducted in an ultrasonicator (Kunshan Ultrasonic Instrument Co., Suzhou, China) under the following conditions: solvent (distilled water)–material ratio, 30:1 (mL/g); extracting temperature, 50 °C; extracting time, 0.5 h; ultrasonic power, 180 W. After extraction, the residue was collected by centrifugation (6000 r/min, 15 min), then precipitated by 90% (*v*/*v*) aqueous ethanol at 4 °C for 24 h. Finally, the sediment was freeze-dried to yield polysaccharides. The CPP generated by HWE, AAE, EAE, HPE, and UAE were named as CPP-W, CPP-A, CPP-E, CPP-P, and CPP-U, respectively.

### 2.3. General Chemical Properties

The polysaccharide extraction yield (%) was calculated as follows: % Yield = [polysaccharides contents of extraction (g)]/[the dry weight of *Citrus reticulata* Blanco cv. Tankan peel powder (g)] × 100%. Polysaccharide content in CPP was determined via the phenol-sulfuric acid assay using D-glucose as the standard [11]. Sulfate radical content in CPP was measured through barium chloride-gelatin turbidimetric nephelometry calibrated against potassium sulfate [12]. Uronic acid in CPP was quantified by the carbazole-sulfuric acid method with galacturonic acid as the external standard [13]. Total protein content in CPP was assessed by Bradford assay using bovine serum albumin as the calibrator.

### 2.4. Molecular Weight Analysis

The molecular weights of various CPPs were quantified by gel permeation chromatography (GPC) using an established protocol with modifications [14]. Chromatographic separation employed a Waters 2414 HPLC system (Waters, Milford, MA, USA) fitted with an Ultrahydrogel™ 250 analytical column (7.8 × 300 mm, 6 μm). Other conditions included oven temperature, 35 °C; mobile phase, K_2_HPO_4_ buffer (0.02 M); flow velocity, 0.5 mL/min; injection volume, 20 μL. Before analyses, the samples (3 mg) were dissolved in 1 mL of deionized water and filtered through a 0.22 μm membrane. The molecular weight of CPPs was calculated using the Astra software (version 6.1.7, Wyatt Technologies Co., Santa Barbara, CA, USA).

### 2.5. Monosaccharide Composition Analysis

The monosaccharide profiles of CPPs were measured based on the modified method [15]. Monosaccharide composition of CPPs was analyzed on an Agilent 1100 HPLC (Santa Clara, CA, USA) with ZORBAX SB-C18 column (4.6 × 250 mm, 5 μm) and DAD detection. Mobile phase comprised phosphate buffer (0.1 M, pH 6.7) (A) and acetonitrile (B). Other HPLC parameters were as follows: flow rate, 1 mL/min; oven temperature, 30 °C; sample size, 5 µL; detection wavelength, 250 nm. Prior to the analytical procedure, the samples (3 mg) underwent hydrolysis with trifluoroacetic acid (4 M) in a hermetically sealed glass vial (110 °C, 8 h). Post-hydrolysis samples underwent dual methanol co-evaporation under N_2_ purge, followed by reconstitution in ultrapure water and syringe filtration (polyvinylidene fluoride, PVDF, 0.22 μm) prior to chromatographic analysis. The monosaccharide standards underwent the same derivatization and analysis procedure.

### 2.6. Molecular Morphology Observation

The molecular morphological characteristics of polysaccharides were characterized using scanning electron microscopy (SEM) and atomic force microscopy (AFM). For SEM, dried CPP samples were mounted on silicon wafers, sputter-coated with gold, and examined by S4800 SEM (Hitachi, Tokyo, Japan). Sample solutions (10 μg/mL) with 5 μL aliquots were deposited onto freshly cleaved mica surfaces. After air-drying for 4 h, AFM characterization was performed using a Multimode 8 system (Bruker Corporation, Berlin, Germany) with a scanning resolution of 256 × 256 pixels [16].

### 2.7. Fourier Transform Infrared Spectrometer (FT-IR) Analysis

Pre-dried CPPs were mixed with KBr (1:100, *w*/*w*) in an anhydrous mortar. The blend was ground to fine particles and pressed into a translucent disk. FT-IR measurements were conducted with a Nicolet iS50 spectrometer (Thermo Fisher Scientific, Waltham, MA, USA) spanning 4000–400 cm^−1^.

### 2.8. Nuclear Magnetic Resonance (NMR) Analysis

For NMR analysis, each CPP specimen (approximately 30 mg) was dissolved in 0.5 mL of D_2_O within an NMR tube, then ^1^H/^13^C NMR data were acquired via an AVANCE NEO 500 MHz instrument (Bruker Corporation, Berlin, Germany) fitted with a TXI probe (5 mm). Chemical shift was referenced in ppm.

### 2.9. Cell Viability Analysis

Cells were cultured (37 °C, 5% CO_2_) in DMEM + 10% FBS (*v*/*v*) + penicillin (100 U/mL) + streptomycin (100 μg/mL). Cell viability following CPP treatment was assessed by the MTT colorimetry, as described previously [17]. Briefly, RAW264.7 macrophages (5 × 10^3^ cells/well) were plated in 96-well microplates and allowed to adhere for 8 h. After removal of the culture medium, cells were treated with CPP (100–400 µg/mL) for 24 h. The untreated cells served as the blank control. Subsequently, MTT solution (100 μL, 0.5 mg/mL) was added to each well followed by 4 h dark incubation at 37 °C. Formazan crystals were then dissolved with 150 μL of dimethylsulfoxide (DMSO), and absorbance was read at 570 nm.

### 2.10. Immune Agents Determination

RAW 264.7 macrophages were plated in 96-well microplates at a density of 5 × 10^5^ cells/well and allowed to adhere for 8 h under standard culture conditions. After removal of the culture medium, cells were interfered with CPP (100–400 µg/mL) for 24 h. The untreated cells and LPS (1 μg/mL)-treated cells served as the blank control and positive control, respectively. The level of nitric oxide (NO) in cell culture supernatant was quantified by the Griess assay, as described previously [17]. The prostaglandin E_2_ (PGE_2_), interleukin-6 (IL-6), tumor necrosis factor-α(TNF-α) concentrations were quantified using ELISA kits (Beyotime, Shanghai, China) per manufacturer’s protocols.

### 2.11. Western Blot

Cell lysates prepared in radio immunoprecipitation assay (RIPA) buffer underwent centrifugation (15,000 r/min, 20 min, 4 °C) with subsequent supernatant collection. Protein quantification was performed using a BCA protein assay kit (Servicebio, Wuhan, China). Proteins (20 μg/lane) were loaded on the 10% sodium dodecyl sulfate- polyacrylamide gel electrophoresis (SDS-PAGE) and transferred onto a PVDF membrane (0.22 μm). Subsequently, membranes were blocked with 5% (*w*/*v*) skim milk in 0.05% (*w*/*v*) tris buffered Saline with tween (TBST) for 1 h at 25 °C, following incubation at 4 °C overnight with the primary antibodies. Membranes were washed thrice with TBST and incubated with specific secondary antibody for 30 min at 25 °C. Signals were developed with an enhanced chem-iluminescence kit (Bio-Rad, Hercules, CA, USA).

### 2.12. Statistical Analysis

Experiments were repeated at least three times, and the results were expressed as mean ± SD. Data was analyzed using ANOVA followed by Duncan’s multiple range test using SPSS 24.0 statistical software (SPSS Inc., Chicago, IL, USA) for statistical significance (*p* < 0.05).

## 3. Results and Discussion

### 3.1. Comparative Analysis of Polysaccharide Yield Among Five Different Methods

The yields of *Citrus reticulata* Blanco cv. Tankan peel polysaccharides (CPP) obtained by diverse methods are shown in Table 1. Compared with the conventional HWE method, the alternative extraction techniques (AAE, EAE, HPE, and UAE) enhanced the CPP yield. The CPP-P (11.75%) yield was prominently (*p* < 0.05) higher than other methods, indicating that HPE could be an effective method to extract CPP from *Citrus reticulata* Blanco cv. Tankan peel. This was consistent with a previous study, which compared the yields of *Laminaria japonica* polysaccharides extracted using different methods [18]. The highest yield of CPP-P may be due to the rapid changes in pressure during high-pressure extraction, inducing the disruption of raw material cells and tissues, resulting in a high polysaccharide dissolution rate [19].

### 3.2. Physical–Chemical Characteristics of the CPPs Extracted by Five Different Methods

As listed in Table 1, 1.64–6.28% of the sulfonic acid group was found in CPPs. Uronic acid is formed by oxidizing monosaccharide hydroxyl groups to carboxyl groups. The uronic acid content of CPPs extracted under different conditions exhibited significant variations (*p* < 0.05). CPP-P (11.65%) possessed the highest uronic acid content, followed by CPP-E (7.52%), while CPP-W (2.88%) had the lowest uronic acid content. The protein contents of the CPPs extracted by different methods were less than 1.49%, suggesting their efficient elimination. Table 1 demonstrates that different extraction methods resulted in polysaccharides with different molecular weights (Mw). CPP-A presented the smallest Mw (1788 kDa), which corresponded with Gao et al. results [18], which showed that *Laminaria japonica* polysaccharides extracted with acid assistance had the lowest Mw compared with other techniques. This may be due to the fact that the glycosidic bonds were broken in acidic extraction environments, which greatly reduced the Mw of polysaccharides [20]. CPP-P had the largest Mw of 11,267 kDa, which may result from polysaccharide aggregation tightly under high-pressure conditions [21]. CPP-W and CPP-E had similar Mw of 5908 kDa and 5264 kDa, respectively.

### 3.3. Surface Morphology of the CPPs Extracted by Five Different Methods

**Figure 2:**
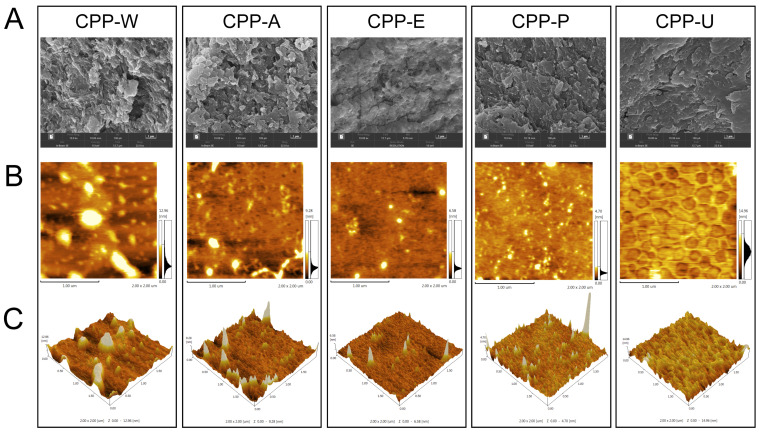
Morphological characterizations of the CPPs extracted by five different methods: (**A**) SEM micrographs (scale bar: 1 μm), (**B**) AFM planar images (scan size: 4 μm^2^), (**C**) AFM 3D surface plots.

The micro-morphology of each CPP was examined using SEM and AFM. Figure 2A shows that the five CPPs exhibited obvious distinction in their morphological characteristics. CPP-W displayed a stratified structure with a porous organization and a distinctly coarse surface texture. Conversely, CPP-A and CPP-P were defined by their porous, multilamellar, and reticulated architectures. The surface of CPP-U, however, was distinguished by its smoothness, density, and scarcity of fissures. These observations suggested that the UAE not only perturbed the plant cell structure but also significantly altered the polysaccharides’ surface topography [22,23]. Figure 2B,C shows that all the CPPs principally displayed interwoven chain-like or reticulated structures with varying degrees of branching, indicating the aggregations and entanglement of branched polysaccharide chains [24]. CPP-U demonstrated a gel-like network structure, whereas CPP-P and CPP-A featured finer protrusions and greater aggregations than CPP-W and CPP-E. This increased aggregation was hypothesized to result from the tight entanglement of side chains, leading to the formation of aggregates [25]. The intermolecular aggregation was facilitated by the interactions between the hydroxyl groups on the polysaccharide side chains and water molecules [26,27]. Furthermore, CPP-P showed a great number of irregular protrusions, indicating a more extensive branched structure, which suggested strong interactions with water molecules. Consequently, the five different extraction methods exerted varying degrees of influence on the main chains and side chains of CPPs, resulting in polysaccharides with different levels of aggregation. The above results demonstrated that different extraction methods significantly influenced the microstructure of CPPs. This was in agreement with previous reports that the polysaccharide’s morphology may be influenced by pretreatment procedures and extraction methods [28,29]. Molecular interactions cannot be conclusively determined from microscopic images alone; thus, further studies are necessary to investigate the structural properties of CPPs by using super-resolution SEM, DSC, and XRD quantitative analysis.

### 3.4. Monosaccharide Compositions of the CPPs Extracted by Five Different Methods

**Figure 3:**
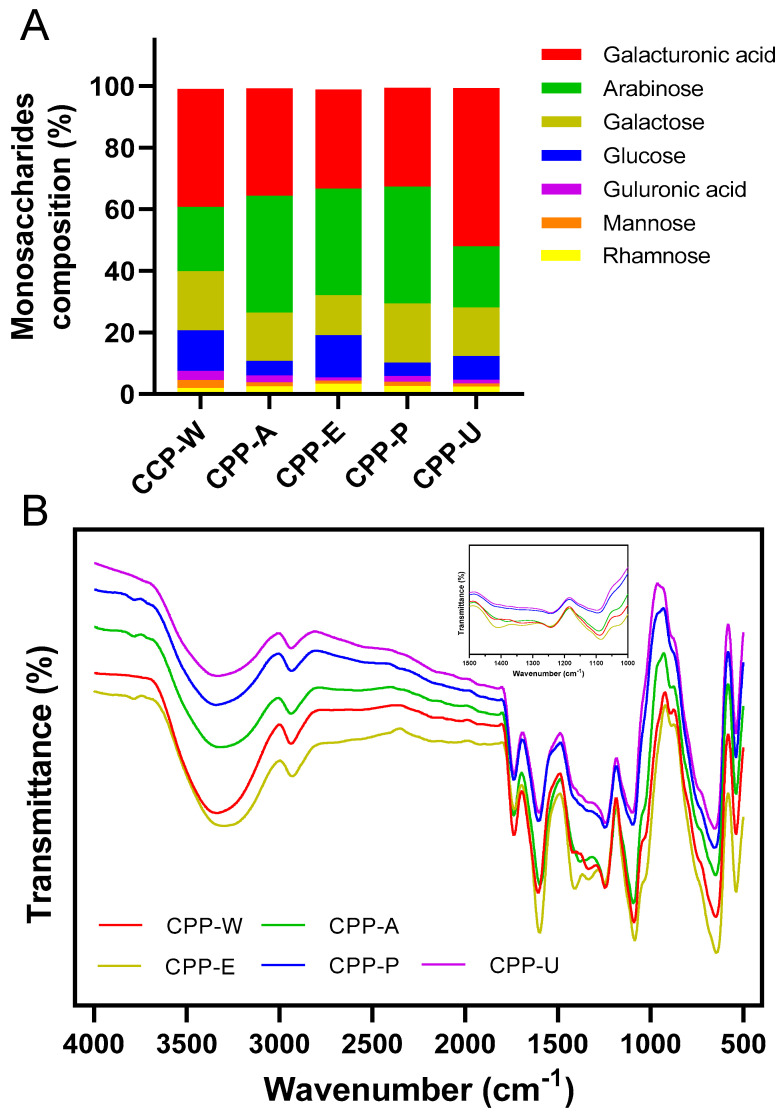
Monosaccharide compositions of the CPPs extracted by five different methods (**A**); FT-IR spectroscopy of the CPPs extracted by five different methods (**B**).

Figure 3A shows that polysaccharides extracted by the five methods had analogous monosaccharide compositions, but different in proportion. Five CPPs were primarily composed of galacturonic acid, arabinose, galactose, and glucose, accounting for more than 80% of the total monosaccharide content. Consistent with this study, Li et al. found that the citrus peel polysaccharides extracted by ultrasound included galacturonic acid, arabinose and galactose [30]. The total proportion of fucosidase, glucuronic acid, ribose, mannuronic acid, and glucosamine was less than 1.2%; thus, their proportions were too low to be visible in Figure 3A. The galacturonic acid content in CPP-W, CPP-A, CPP-E, CPP-P, and CPP-U was 38.31%, 34.92%, 32.21%, 32.08%, and 51.41%, respectively, which confirmed that five CPPs were classified as acidic hetero-polysaccharides. Furthermore, the high contents of arabinose and galactose were observed in CPP-A and CPP-P, which may be attributed to acid and high-pressure treatments that facilitated the hydrolysis of the polysaccharide chains [19]. All five CPPs contained a few guluronic acid levels, and CPP-W contained the highest proportion of guluronic acid, followed by CPP-A. The findings demonstrated that elevated temperature and acidic conditions promoted the extraction of guluronic acid from *Citrus reticulata* Blanco cv. Tankan peel, which was aligned with Tang et al. [31], who compared the polysaccharides derived from banana flower using diverse methods. Therefore, the data explained that extraction methods significantly altered the monosaccharide compositions of CPPs.

### 3.5. FT-IR Spectroscopy of the CPPs Extracted by Five Different Methods

The structural properties of CPPs were examined by employing FT-IR spectroscopy. Figure 3B shows that the five CPPs exhibited analogous spectral characteristics, with minor variations in transmittance and wavenumber of some characteristic bands, indicating similar chemical structures. As listed in Table 2, the peak at 3351 cm^−1^ was due to the stretching vibration of O-H, and the peak at 2930 cm^−1^ corresponded with the stretching vibration of C-H, which was the characteristic absorption of polysaccharides [32]. The peak of CPPs near 1733 cm^−1^ was due to the C=O stretching vibration of methyl-esterified COO− moiety [33]. The band of CPPs at 1592 cm^−1^ was ascribed to the carbonyl group of galacturonic acid. Additionally, the band detected at 1230 cm^−1^ was attributed to the S=O asymmetric stretching vibration of sulfate radicals [34], aligning with the chemical component analysis. The three signals at 1090 cm^−1^, 896 cm^−1^, and 830 cm^−1^ confirmed that the five CPPs were mainly composed of α-pyranose [35]. The results revealed that the five extraction techniques had no significant impact on the glycosidic bond configurations or major functional groups of CPPs. As indicated by the FT-IR analysis, the characteristic organic groups of CPPs extracted by different methods had no significant differences. Future studies will employ high-resolution FT-IR with increased scans to better resolve subtle structural differences among the CPPs.

### 3.6. NMR Spectra of the CPPs Extracted by Five Different Methods

**Figure 4:**
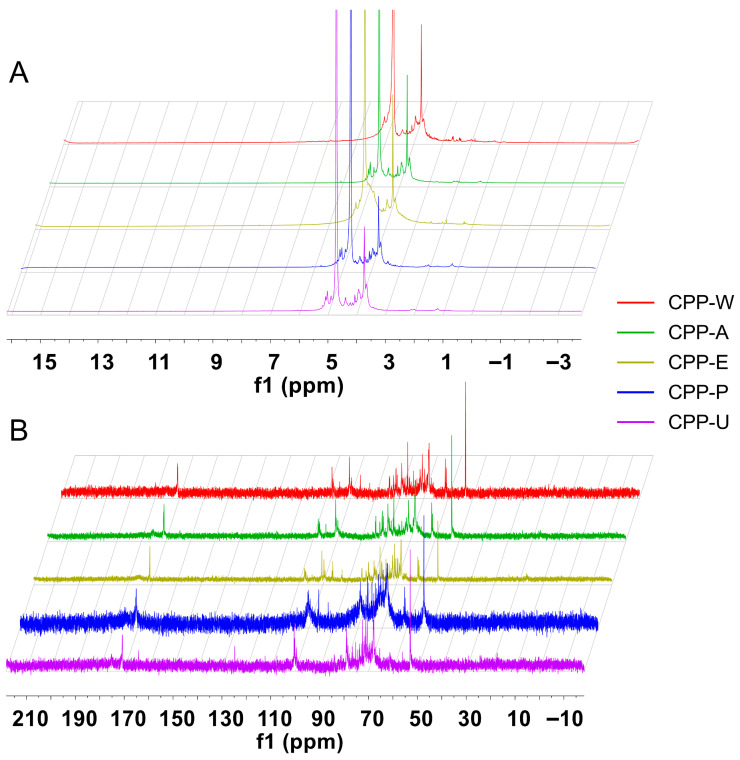
^1^H NMR spectra (**A**) and ^13^C NMR spectra (**B**) of CPPs extracted by five different methods.

Most of the proton signals of all five CPPs appeared in the region of δ H 3.0–5.5 ppm (Figure 4A) and δ C 60–110 ppm (Figure 4B), which were the typical NMR patterns of polysaccharides [36]. The observation of anomeric proton resonances (4.0–5.1 ppm) and anomeric carbon signals (90–110 ppm) confirmed the coexistence of both α- and β-anomeric configurations within the CPPs [37]. The signal of D_2_O (δ H 4.7 ppm) obscured some anomeric proton signals. The signals of all five CPPs at δ H 5.1–5.8 ppm and δ C 98–103 ppm in the NMR spectrum were attributed to the anomeric proton and anomeric carbon of the α-type glycosidic bond, respectively [38]. This indicated the ubiquity of this glycosidic bond type in the extracted polysaccharides regardless of the method used. Additionally, the signals of all five CPPs at δ H 4.3–4.8 ppm and δ C 103–106 ppm in anomeric regions were due to the β-configurations [39]. Figure 4B shows that the abundance of ^13^C NMR peaks of CPP-W, CPP-P and CPP-U were obviously lower than that of CPP-A and CPP-E, reflecting differential glycosidic linkage ratios among the CPPs. The relatively weak signal at δ C 100 ppm of all five CPPs were due to the C-1 of α-D-Manp [40]. The signals of all five CPPs at near δ C 170 ppm displayed the existence of carboxyl carbon, which confirmed the conclusion of infrared spectroscopy analysis that CPPs were composed of galacturonic acid [41]. Overall, the different extraction methods influenced the polysaccharide chain length of CPPs without altering fundamental architecture, which was consistent with the FT-IR results.

### 3.7. Effects of the CPPs Extracted by Five Different Methods on RAW264.7 Cell Viability

**Figure 5:**
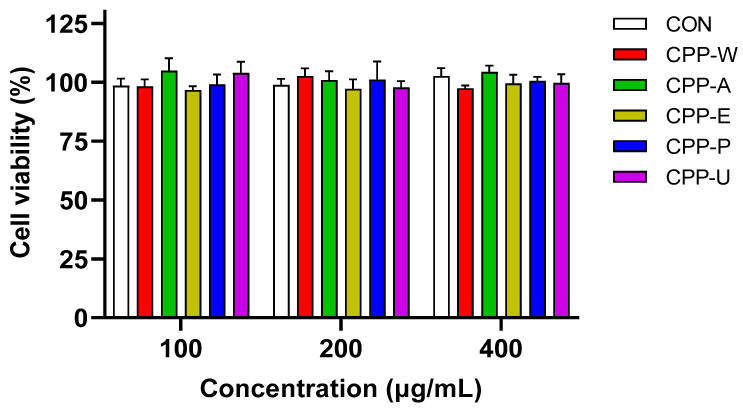
Cytotoxicity evaluations of the CPPs extracted by five different methods in RAW264.7 macrophages.

Polysaccharides enhance immunity primarily through direct activation of innate immune cells, which serve as crucial immunoregulatory mediators in host defense [42,43]. Accordingly, this study compared the immunostimulatory efficacy of different CPPs on RAW264.7 macrophages. The cytotoxic effects of five distinct CPPs were primarily evaluated through MTT colorimetric analysis. Figure 5 shows that CPPs extracted by the five methods were found to be non-cytotoxic within 100–400 μg/mL. Therefore, these concentrations were employed for the subsequent research.

### 3.8. Effects of the CPPs Extracted by Five Different Methods on Immune Agents in RAW264.7 Cells

**Figure 6:**
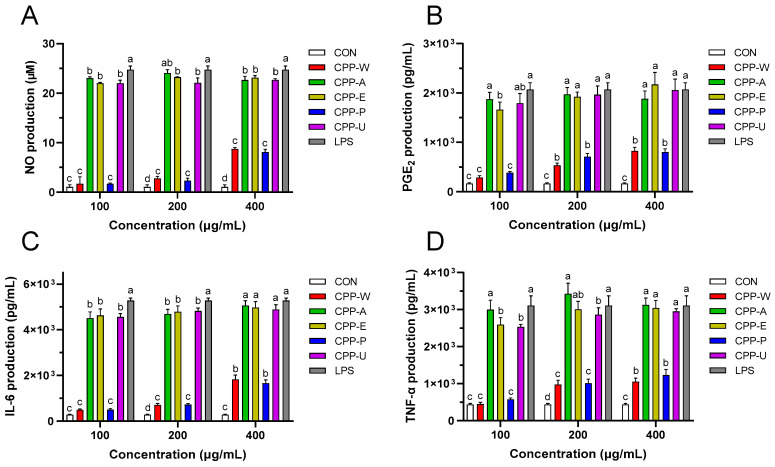
Modulations of immune mediators including NO (**A**), PGE_2_ (**B**), IL-6 (**C**), and TNF-α (**D**) by the CPPs extracted by five different methods in RAW264.7 cells. Cells were treated with or without CPP (100–400 μg/mL) for 24 h. The untreated cells and LPS (1 μg/mL)-treated cells served as the blank control (CON) and positive control (LPS), respectively. Distinct letters within columns indicate significant differences (*p* < 0.05).

Activation of macrophages, a critical component of innate immunity, is closely linked to immunomodulator production [44]. Nitric oxide (NO) potentiates innate immune responses by exerting direct antimicrobial effects against pathogens [45]. Prostaglandin E_2_ (PGE_2_) functions as a pivotal immunomodulatory mediator by orchestrating host defense mechanisms against diverse pathogens, including bacteria, fungi, and viruses [46]. Interleukin-6 (IL-6) modulates adaptive immunity by inducing lymphocyte differentiation and concurrently enhances macrophage phagocytosis [47]. Tumor necrosis factor-α (TNF-α) mediates host defense during acute infections by enhancing macrophage phagocytic activity to eliminate pathogens [48]. Therefore, this study monitored the influences of CPPs on NO, PGE_2_, IL-6, and TNF-α productions in RAW264.7 cells. Figure 6A indicated that the generation of intracellular NO was prominently (*p* < 0.05) enhanced by five CPPs. All five polysaccharides at 200 and 400 μg/mL exhibited significant NO release-promoting activities. Analogous patterns (Figure 6B–D) were identified in the PGE_2_, MCP-1, and TNF-α analysis, indicating five CPPs had immuno-enhancing activities. A previous study has uncovered that the immunological activity of polysaccharides was significantly determined by their skeletal structure, with a high level of negatively charged carboxyl groups from galacturonic acid residues [49]. Therefore, the strong immuno-promoting abilities of CPPs depended on their high proportion of galacturonic acid. These results aligned with high-galacturonic acid *Platycodon grandiflorum* polysaccharides in enhancing NO, IL-6, and TNF-α productions [50]. At concentrations of 100 μg/mL, CPP-A, CPP-E and CPP-U were superior to CPP-W and CPP-P in promoting the release of immune agents. This may be attributed to the fact that low-MW polysaccharides CPP-A, CPP-E, and CPP-U exhibited strong affinity for phagocytes, thereby facilitating immune activation [51].

### 3.9. Effects of the CPPs Extracted by Five Different Methods on Immune-Specific Protein Expression in RAW264.7 Cells

**Figure 7:**
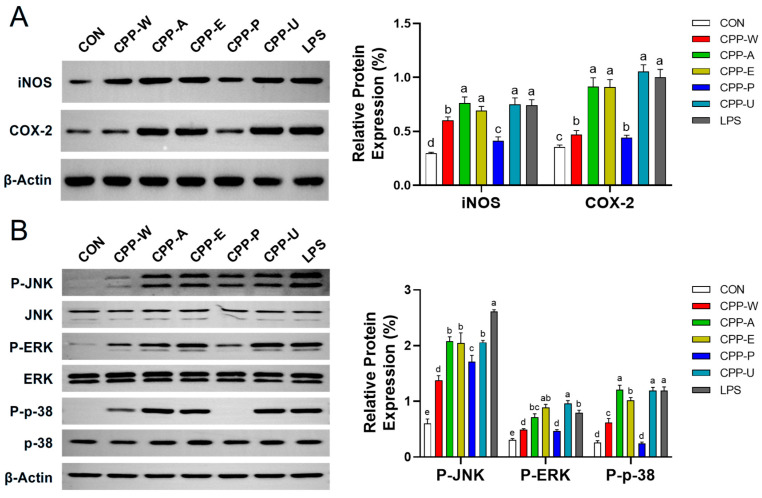
Modulations of iNOS and COX-2 expressions by the CPPs extracted by five different methods in RAW264.7 macrophages (**A**). Modulations of MAPKs (ERK/JNK/p38) by the CPPs extracted by five different methods in RAW264.7 cells (**B**). Cells were intervened with or without CPP (100 μM) for 24 h (iNOS and COX-2) or 1 h (MAPKs). The untreated cells and LPS (1 μg/mL)-treated cells served as the blank control (CON) and positive control (LPS), respectively. Distinct letters within columns indicate significant differences (*p* < 0.05).

The catalytic activity of nitric oxide synthase (iNOS) facilitates L-arginine conversion to NO, a critical mediator augmenting phagocytic capacity and intracellular microbe eradication [43]. Concurrently, cyclooxygenase-2 (COX-2)-mediated metabolism of arachidonic acid yields PGE_2_, a pivotal regulator of gastrointestinal immune homeostasis [52]. This study further investigated whether the impacts of CPPs on NO and PGE_2_ productions were mediated through the corresponding iNOS and COX-2. Figure 7A shows that five CPPs (100–400 μg/mL) treatments markedly (*p* < 0.05) increased iNOS and COX-2 expressions in RAW264.7 cells. This was consistent with a prior report, which demonstrated that polysaccharides extracted from *Tetrastigma hemsleyanum* could promote the generation of immune factors (NO, IL-6, and TNF-α) by up-regulating iNOS and COX-2 expressions [53]. The experimental data demonstrated that CPPs obtained through the five extraction techniques promoted the secretions of immune agents by enhancing iNOS and COX-2 expressions.

### 3.10. Effects of the CPPs Extracted by Five Different Methods on MAPK Pathway in RAW264.7 Cells

The mitogen-activated protein kinases (MAPKs: ERK1/2, JNK, p38) act as the upstream regulators of iNOS and COX-2 and critically govern innate immune responses through modulating immune cell activation, proliferation, and differentiation [54]. Thus, this study further explored the effects of five CPPs on ERK, JNK, and p38 phosphorylation in macrophages. Figure 7B presents significant regulation of ERK1/2, JNK, and p38 phosphorylations by all five CPPs. In comparison, CPP-A, CPP-E, and CPP-U were superior to CPP-W and CPP-P in activating MAPKs in macrophages. Wang et al. [55] also found that *Flammulina velutipes* polysaccharides promoted the secretions of immune factors (IL-6, IL-1β, and TNF-α) via activation of the MAPK signaling pathway to exert immunomodulatory function in RAW264.7 cells. Therefore, the above findings elucidated that the CPPs (especially CPP-A, CPP-E, and CPP-U) might achieve their immunoregulation capacities by activating the MAPK signaling pathway-mediated up-regulation of iNOS and COX-2. Based on these findings, this study provided valuable insights for the future application of CPPs. The differential structural characteristics and bioactivities observed under various extraction methods offered a foundation for selecting appropriate CPPs for specific industrial uses. For instance, CPPs with high immunological potency (such as CPP-A, CPP-E, and CPP-U) showed great potential as natural immunomodulatory agents in functional foods and pharmaceutical formulations. In particular, the enzyme-extracted polysaccharide (CPP-E), which combined high yield, low molecular weight, and significant immunoactivity, represented a promising candidate for scalable and environmentally friendly extraction processes. Future studies should focus on process optimization at pilot scale, toxicological evaluation, and in vivo validation to facilitate commercial applications in the health product and nutraceutical industries.

## 4. Conclusions

This study explored how five extraction methods (HWE, AAE, EAE, PAE, and UAE) influenced the yield, chemical property, molecular structure, and immunostimulatory activity of CPP. Among these, AAE and HPE demonstrated superior extraction yields. Structural analyses revealed distinct chemical properties, molecular weights, monosaccharide compositions, and surface morphologies among the five CPPs, though their core glycosidic linkages and infrared spectral characteristics remained consistent, indicating preservation of the primary polysaccharide framework despite method-specific degradation effects. Notably, CPP-A, CPP-E, and CPP-U exhibited enhanced immunomodulatory activities, attributed to their elevated galacturonic acid content and reduced molecular weight. Mechanistically, CPPs were shown to prominently facilitate the secretions of immune cytokines by activating the downstream iNOS and COX-2 related MAPK pathway. Combined, CPP-E was extracted with high yield, low molecular weight, and robust immune-enhancing properties. These findings collectively established EAE as the optimal method for CPP extraction, offering a strategic foundation for industrial-scale production and functional applications of *Citrus reticulata* Blanco cv. Tankan peel polysaccharides in immunomodulation. Future studies should elucidate the in-depth mechanisms by which these polysaccharides exert immune-promoting effects in vivo.

## Figures and Tables

**Table 1:** Extraction yields and chemical compositions of CPPs extracted from *Citrus reticulata* Blanco cv. Tankan by five different methods.

Samples	CPP-W	CPP-A	CPP-E	CPP-P	CPP-U
**Polysaccharide yield (%)**	2.13 ± 0.02 ^e^	3.66 ± 0.05 ^d^	9.34 ± 0.02 ^b^	11.75 ± 0.05 ^a^	4.88 ± 0.03 ^c^
**Sulfuric radical (%)**	4.20 ± 0.03 ^d^	1.64 ± 0.01 ^e^	4.57 ± 0.03 ^c^	6.28 ± 0.03 ^a^	5.85 ± 0.01 ^b^
**Uronic acid (%)**	2.88 ± 0.18 ^e^	6.91 ± 0.05 ^c^	7.52 ± 0.29 ^b^	11.65 ± 0.26 ^a^	5.18 ± 0.26 ^d^
**Protein (%)**	1.02 ± 0.02 ^b^	0.71 ± 0.03 ^c^	1.00 ± 0.02 ^b^	1.48 ± 0.04 ^a^	1.49 ± 0.00 ^a^
**Mw (kDa)**	5908	1788	5264	11,267	4535

Data are expressed as means ± SD (n = 3). Distinct superscript letters within rows indicate significant differences (*p* < 0.05). Polysaccharide yield (%, *w*/*w*) = [polysaccharides contents of extraction (g)]/[the dry weight of *Citrus reticulata* Blanco cv. Tankan peel powder (g)] × 100%. Chemical composition (except yield) (%, *w*/*w*) = substance/polysaccharide weight. Mw: weight-average molecular weight.

**Table 2:** FT-IR characteristic peaks of CPPs extracted by five different methods.

Wavenumber (cm^−1^)	Assignment	Vibration Mode	References
3351	O-H	Stretching vibration	[31]
2930	C-H	Stretching vibration	[31]
1733	C=O, methyl-esterified	Stretching vibration	[32]
1592	C=O, galacturonic acid	Stretching vibration	[33]
1230	S=O, sulfate	Asymmetric stretching vibration	[33]
1090	pyranose ring/C-O-C	Stretching vibration	[34]
896	α-glycosidic linkage	Deformation vibration	[34]
830	α-pyranose	Ring vibration	[34]

## Data Availability

The original contributions presented in the study are included in the article, further inquiries can be directed to the corresponding authors.
